# *FocVel1* influences asexual production, filamentous growth, biofilm formation, and virulence in *Fusarium oxysporum* f. sp. *cucumerinum*

**DOI:** 10.3389/fpls.2015.00312

**Published:** 2015-05-06

**Authors:** Peiqian Li, Xiaoming Pu, Baozhen Feng, Qiyun Yang, Huifang Shen, Jingxin Zhang, Birun Lin

**Affiliations:** ^1^Key Laboratory of New Techniques for Plant Protection in Guangdong, Institute of Plant Protection, Guangdong Academy of Agricultural SciencesGuangzhou, China; ^2^Department of Life Sciences, Yuncheng UniversityYuncheng, China; ^3^Department of Plant Pathology, College of Agriculture, Guangxi UniversityNanning, China

**Keywords:** *Fusarium oxysporum* f. sp. *cucumerinum*, velvet protein, adherence, biofilm, virulence

## Abstract

Velvet genes play critical roles in the regulation of diverse cellular processes. In current study, we identified the gene *FocVel1*, a homolog of *Fusarium graminearum* VelA, in the plant pathogenic fungus *F*. *oxysporum* f. sp. *cucumerinum*. This pathogen causes the destructive disease called cucumber *Fusarium* wilt (CFW), which severely affects the production and marketing of this vegetable worldwide. Transcript analyses revealed high expression of *FocVel1* during conidiophore development. Disruption of the *FocVel1* gene led to several phenotypic defects, including reduction in aerial hyphal formation and conidial production. The deletion mutant Δ*FocVel1* showed increased resistance to both osmotic stress and cell wall-damaging agents, but increased sensitivity to iprodione and prochloraz fungicides, which may be related to changes in cell wall components. In the process of biofilm formation *in vitro*, the mutant strain Δ*FocVel1* displayed not only a reduction in spore aggregation but also a delay in conidial germination on the polystyrene surface, which may result in defects in biofilm formation. Moreover, pathogenicity assays showed that the mutant Δ*FocVel1* exhibited impaired virulence in cucumber seedlings. And the genetic complementation of the mutant with the wild-type *FocVel1* gene restored all the defects of the Δ*FocVel1*. Taken together, the results of this study indicated that *FocVel1* played a critical role in the regulation of various cellular processes and pathogenicity in *F*. *oxysporum* f. sp. *cucumerinum*.

## Introduction

The cucumber (*Cucumis sativus* L.) is one of the most common vegetables worldwide. However, the quality and productivity of this plant is often threatened by cucumber Fusarium wilt (CFW), a devastating soil-borne vascular fungal disease caused by *Fusarium oxysporum* f. sp. *cucumerinum* (Zhao et al., 2012). To date, efficient strategies for the management of Fusarium wilt have not been developed, which could be explained in part by our limited information regarding the biology of *F. oxysporum* f. sp. *cucumerinum*.

Biofilm formation is one of the most common mechanisms of growth in microorganisms, and it often displays altered phenotypes with respect to growth rate, gene transcription, and resistance to various stresses (Wosten et al., 1999; Harding et al., 2009; Mowat et al., 2009). Biofilms are dense, highly hydrated cell clusters that form on surfaces and are embedded in a self-produced gelatinous matrix comprising extracellular polymeric substances (EPSs) (Blankenship and Mitchell, 2006; Harding et al., 2009). Biofilm formation is a well-organized process, which depends on surface properties, conditioning films on the surface, characteristics of the medium, and microbial cell properties (Donlan, 2002). Moreover, the formation, maturation, and dispersal of biofilms are key processes in the life cycle of many animal and plant bacterial pathogens (Costerton et al., 1995; O'Toole et al., 2000).

In an earlier study by our group, *F. oxysporum* f. sp. *cucumerinum* was found to form biofilms in flat-bottomed polystyrene microtitre plates and was less susceptible to environmental stresses than planktonic counterparts (Li et al., 2014). Biofilm formation by filamentous fungi has been described for *Aspergillus niger* grown on polyester mesh squares (Villena and Gutierrez-Correa, 2003), and for *F. solani* and *F. oxysporum* (Imamura et al., 2008; Li et al., 2014). Fungal biofilm formation comprises three basic time-dependent phases: (i) adhesion, which is strongly increased by spore hydrophobicity; (ii) initial growth and development, from spore germination to surface colonization, which is affected by inoculum density; and (iii) maturation wherein biomass density increases (Harding et al., 2009; Mowat et al., 2009).

Cell-substrate interactions and cell-cell adherence represent the basis for the formation of fungal biofilms (Harding et al., 2009). In pathogenic fungi, spore adherence to the host surface is usually a prerequisite for infection (Priegnitz et al., 2012). Consequently, maybe the spore adhesion and filamentation in fungi are prerequisites for robust biofilm development and virulence. Biofilm formation not only represents a mere biological coating but also provides important clues for determining appropriate therapeutic strategies against certain microbes (Harding et al., 2009). Therefore, a better understanding of the regulatory mechanisms of biofilm formation and virulence will be essential to facilitate the development of efficient control strategies against CFW.

The veA family of velvet proteins is conserved throughout the fungal kingdom (Li et al., 2006), and has been proven to be involved in regulating diverse cellular processes, including control of conidial differentiation, hyphal hydrophobicity, and secondary metabolism in several fungal species (Bayram et al., 2008; Calvo, 2008). Recently, the functions of VeA have been investigated in several other filamentous fungi including *Acremonium chrysogenum* (Dreyer et al., 2007), *Fusarium verticillioides* (Li et al., 2006; Myung et al., 2009), *Mycosphaerella graminicola* (Choi and Goodwin, 2011), *Penicillium chrysogenum* (Hoff et al., 2010), and *F. graminearum* (Jiang et al., 2011). In these species, VeA deletion mutants present different phenotypic characteristics. For example, deletion of the *VEA* gene (*FvVE1*) in *F. verticillioides* suppresses aerial hyphal growth and reduces colony surface hydrophobicity on solid media. The deletion of *FgVeA* affects hyphal differentiation, conidial germination, and cell wall integrity in *F. graminearum* (Jiang et al., 2011). The *FfVel1* deletion mutant in *F. fujikuroi* failed to regulate the biosynthesis of gibberellins and fusarin, and did not affect rice seedlings infection (Wiemann et al., 2010). Thus, VeA might be involved in various physiological mechanisms in different fungal species. Since the VeA protein plays a key role in filamentation and the hydrophobic properties of the cell surface in some filamentous fungi (Li et al., 2006; Jiang et al., 2011), we hypothesized that these proteins would function as a core component of the velvet complex and would regulate biofilm formation in *F. oxysporum* f. sp. *cucumerinum*. Therefore, in this study, we deleted *FocVel1* in *F. oxysporum* f. sp. *cucumerinum* and analyzed the phenotypes of wild-type and deletion mutant strains. All results indicated that *FocVel1* played a critical role in conidial production, aerial hyphal formation, biofilm formation, and pathogenicity in *F*. *oxysporum* f. sp. *cucumerinum*.

## Materials and methods

### Strain and culture conditions

The *F. oxysporum* f. sp. *cucumerinum* wild-type isolate Foc-GD (CCTCC AF 2013029) was used in all experiments. Fungal strains were stored as microconidial suspensions in 30% glycerol at −80°C. The wild-type strain and transformants generated in this study were cultured on potato dextrose agar (PDA) or minimal medium (MM) for testing the mycelial growth (Zheng et al., 2013). For conidial production and extraction of genomic DNA, cultures were grown in potato dextrose broth (PDB) at 28°C for 5 days on a rotary shaker (150 rpm) (López-Berges et al., 2013). For biofilm formation, strains were incubated in Sabouraud dextrose broth (SDB; Difco Laboratories, Detroit, MI, USA). Conidia were then harvested by filtration through three layers of sterile gauze and washed with phosphate-buffered saline (PBS) (Li et al., 2014).

### Sequence analysis of *FocVel1*

The velvet protein gene *VeA* (FOXG_11273) was identified in the genome sequence of *F*. *oxysporum* f. sp. *lycopersici* race 2 wild-type isolate 4287 (available at http://www.broadinstitute.org/annotation/genome/fusarium_group/MultiHome.html) (López-Berges et al., 2013). The *F. oxysporum* f. sp. *cucumerinum* VeA homolog, *FocVel1*, was amplified with the primer pair *Focvel*1-F/*Focvel*1-R from the strain Foc-GD. Fungal genomic DNA was extracted from mycelia using an extraction kit (TaKaRa Biotech, China) according to the manufacturer's instructions. A series of primers were designed using the Primer Express 3.0 software according to the identified sequences of FOXG_11273 in the database (Table 1). Polymerase chain reaction (PCR) amplification was performed under the following cycling conditions: initial denaturation at 95°C for 3 min; 35 cycles of denaturation at 94°C for 30 s, annealing at 54°C for 40 s, extension at 72°C for 2 min; and final extension at 72°C for 10 min. The resulting PCR products were purified, cloned and sequenced.

**Table 1:** **Primers used for PCR in this study**.

**Primer name**	**Sequence (5′—3′)**
*FocVel*1-F	TTCATGCGTCTTCCGTTATTCCA
*FocVel*1-R	TCTGATTGATAGGCTTGACTTGAC
73UP1	AAAACTGCAGCGGGGAATAAAGCCACCATC
73UP2	ACCGGAATTCTATTAGTGAGGCGCGTGAGA
73DN3	ACGCGTCGACATCAGCTCCGTCCAGTTCAA
73DN4	GGGGTACCTTGGTTTGGTCTGGTCTGGT
73g-F	TAGTGTGGAAGAGGGCAAGG
73g-R	TGCTTTCCCGCTTCCTCATA
Vel1-CF	CGGGGTACCCTCACTATTCAAACTCATTC
Vel1-CR	CCATCGATAGGTATCTACTCGTCATAATA
Vel1-RTF	CCTCCCGGCTCTCAAGATAG
Vel1-RTR	TAACCACGGTGAGATCGACC
EF-F	CGCTCTTCTTGCCTACACCC
EF-R	ATCTCACGCTCCCAACCCTT

### Quantitative reverse transcription PCR (qRT-PCR) analysis

RNA samples were isolated from mycelia of each strain harvested after 24, 36, 48, 60, and 72 h growth in PDB medium with TRIzol reagent (Invitrogen, Carlsbad, CA, USA) and purified with a DNA-free kit (Ambion). With the M-MLV reverse transcriptase (Invitrogen), first-strand cDNA was synthesized, and quantitative reverse transcription PCR (qRT-PCR) was performed with an ABI 7500 sequence detection system (Applied Biosystems, Foster City, CA, USA) using QuantiTect SYBRGreen PCR Master Mix (Qiagen, China). The *F. oxysporum* translation elongation factor (*EF-1α*) gene was used as the endogenous reference gene for normalization (Gravelat et al., 2010). The primer pairs Vel1-RTF/Vel1-RTR and EF-F/EF-R were used for quantitative RT-PCR (qRT-PCR) analysis (Table 1). The relative expression levels of each transcript were estimated by the 2^−ΔΔCt^ method (Livak and Schmittgen, 2001). All reactions were conducted in triplicate for each sample, and the experiment was repeated thrice.

### Generation of *FocVel1* deletion mutants

The deletion vector pKOV21-*FocVel1*-Del was constructed by inserting the two flanking sequences of *FocVel1* into two sides of the hygromycin resistance (*hph*) gene in the pKOV21 vector. The upstream flanking sequence fragment of *FocVel1* was amplified using the primer pair 73UP1/73UP2, and the 1250-bp amplicon was inserted into the *Pst*I–*EcoR*I site upstream of the *hph* cassette on the pKOV21 vector. Subsequently, the 1270-bp downstream flanking sequence fragment of *FocVel1* was amplified with the primer pair 73DN3/73DN4, and cloned into the *Sal*I–*Kpn*I site downstream of the *hph* cassette. Finally, the resulting *FocVel1* deletion vector pKOV21-*FocVel1*-Del was transformed into protoplasts of Foc-GD according to a previously published protocol (Hou et al., 2002). Hygromycin was added to a final concentration of 100 μg/mL for transformant selection. Putative *FocVel1* deletion mutants were then identified by PCR assays with the primer pair 73g-F/73g-R (as shown in Table 1) and further confirmed by Southern blot hybridization (Chi et al., 2009).

### Complementation of the *FocVel1* gene deletion mutant

To confirm that the phenotype changes in mutants were as a result of the gene deletion, the deletion mutant Δ*FocVel1*-3 was complemented with full-length *FocVel1*. For construction of the complement plasmid pKN-*FocVel1*-C, the full-length *FocVel1* gene, including the 1228-bp upstream promoter region and the 660-bp downstream terminator region, was amplified from genomic DNA of Foc-GD with primer set Vel1-CF/Vel1-CR (Table 1). *FocVel1* gene in the vector was sequenced, and the resulting construct was then co-transformed into protoplasts of the target mutant, as described above, except that G418 (100 μg/mL) was used as a selection agent. Transformants showing resistance to both G418 and hygromycin were selected, screened by PCR for the presence of the complementation construct, and further confirmed by Southern blot analyses. The complemented strains were designated as *ΔFocVel1*-3C.

### Mycelial growth and conidiation assays

The wild-type strain Foc-GD, deletion mutant Δ*FocVel1*-3, and complemented strain Δ*FocVel1*-3C were routinely cultured on PDA or minimal medium (MM) plates at 28°C (Zheng et al., 2013). For testing sensitivities to various stresses, mycelial growth was assayed on PDA supplemented with sorbitol, KCl, iprodione, prochloraz, hymexazol, caffeine, and Congo red (Jiang et al., 2011; Zheng et al., 2013). Each plate (*n* = 3 plates per treatment) was inoculated with a 5-mm-diameter mycelial plug taken from the edge of a 5-day-old colony. After 5 days of incubation at 28°C in the dark, the colony diameter in each plate was measured and the original mycelial plug diameter (5 mm) was removed from each measurement. For the controls, each strain was inoculated on PDA without supplements. The percentage of mycelial growth inhibition (RGI) was calculated using the formula RGI = ([A – B]/A) ×100, where A is the colony diameter of the control, and B is the colony diameter in the test treatment. Each experiment was independently repeated thrice. For conidiation assays, five mycelial plugs (5 mm in diameter) taken from the periphery of a 5-day-old colony of each strain were added to a 150-mL flask containing 70 mL of PDB medium (*n* = 3 flasks per strain). The flasks were incubated at 28°C for 5 days with shaking (150 rpm). The number of conidia in each flask was determined with a hemacytometer and microscope. The experiment was repeated three times.

### Fungal biofilm development assays

Biofilm formation of the wild-type strain Foc-GD and target mutants was determined on the basis of the filamentous, biofilm-forming fungi as reported previously (Li et al., 2014). Flat-bottomed, 96-well, polystyrene microtiter plates (Fisher, Waltham, MA, USA) were inoculated with 200 μL of SDB containing 1 × 10^6^ conidia/ml and incubated for 48 h at 28°C statically to allow the conidia to settle and adhere to the bottom of the plate.

A semiquantitative measurement of *F. oxysporum* f. sp. *cucumerinum* biofilm formation was obtained from the 2,3-bis(2-methoxy-4-nitro-5-sulfophenyl)-2H-tetrazolium-5-carboxanilide (XTT) reduction assay, as described previously (Chandra et al., 2001). Fungal mitochondrial dehydrogenase activity reduces the XTT tetrazolium salt to XTT formazan, resulting in colorimetric change that correlates with cell viability (Meshulam et al., 1995). The colorimetric change was measured using a microtiter reader (Labsystems Multiskan MS; Labsystems, Finland) at 492 nm. For time-series development analysis, the standardized spore suspension was inoculated on 1.5-cm^2^ polystyrene strips (Fisher) for 2, 4, 8, 12 or 24 h, respectively. All assays were carried out in triplicate for each sample.

### Microscopic examination

Hyphal morphology, conidial morphology, and time-dependent formation of biofilm were examined for each strain by fluorescence microscopy (ZVS-47E microscope; Carl Zeiss, Inc., Oberkochen, Germany). The architecture of biofilms was observed using a Zeiss LSM710 confocal laser-scanning microscope (CLSM) equipped with argon and HeNe lasers and mounted on a Zeiss Axiovert 100 M microscope (Carl Zeiss Microscopy GmbH, Hamburg, Germany) following previously described methods (Chandra et al., 2008; Mukherjee et al., 2012). Briefly, after different incubation times, polystyrene strips with biofilms were washed twice with PBS and stained with SYTO-9 (LIVE/DEAD BacLight Bacterial Viability kit; Life Technologies) for 1.5–2 h at 30°C in the dark; heat-killed mature biofilms were then incubated with 2 mL of PBS containing propidium iodide (PI, LIVE/DEAD BacLight Bacterial Viability kit) and concanavalin A-Alexa Fluor 488 conjugate (ConA; 25 μg/mL, Life Technologies) according to the manufacturer's instructions. SYTO-9 is a green-fluorescent nucleic acid stain that generally labels both live and dead cell. PI is a red fluorescent nucleic acid stain that only penetrates cells with damaged membranes, and ConA binding to glucose and mannose residues of cell wall polysaccharides emits green fluorescence.

### Pathogenicity assay on cucumber seedlings

After incubation in PDB medium for 5 days, the conidia of each strain were collected and resuspended in sterilized water to a concentration of 1 × 10^6^ conidia/mL subsequently. Cucumber seedling root inoculation assays were performed as previously described (Pu et al., 2014). Generally, the cucumber seeds (susceptible cultivar, *Cucumis sativus* L. 9930) were surface-disinfected with 2% sodium hypochlorite for 10 min, rinsed with sterile distilled water, and incubated at 28°C for accelerating germination. Second, germinating seeds were selected for growth in hydropnic chambers containing 50% Murashige and Skoog medium (MS) with vitamin supplements (Sigma). When the seedlings at the two-true leaves stage, the roots were wounded and subsequently inoculated with the standard conidial suspension. After inoculation, the seedlings were cultivated in a growth chamber with a 16/8-h day/night schedule at 28°C. At 15 days postinoculation (dpi), the disease severity index (DSI) was calculated as previously described (Pu et al., 2014). Each treatment consisted of three replicates, with 15 seedlings for each replicate.

### Statistical analysis

All experiments were performed in triplicate. Data analysis was performed using SIGMA PLOT 11.0 (http://www.sigmaplot.com). Statistical analysis was evaluated by analysis of variance with the software SPSS 16.0. A *P*-value of < 0.05 was considered statistically significant.

## Results

### Identification of *FocVel1* in *F. oxysporum* f. sp. *cucumerinum*

First, we analyzed the *FocVel1* gene in *F. Oxysporum* f. sp. *cucumerinum*. RT-PCR analysis revealed an open reading frame (ORF) of 1596 bp interrupted by a 94-bp intron. The gene encodes a predicted protein of 532 amino acids and was designated *FocVel1* (GenBank Accession number: KJ716229). The predicted amino acid sequence of *FocVel1* shared 97.56, 97.74, and 78.29% identity with *FfVel1* from *F. fujikuroi* (GenBank: FN548142), *FvVe1* from *F. verticillioides* (GenBank: DQ274059), and *FgVEA* from *F. graminearum* (GenBank: JN635273), respectively (Figure S1).

### Targeted deletion of *FocVel1* gene and complementation

**Figure 1:**
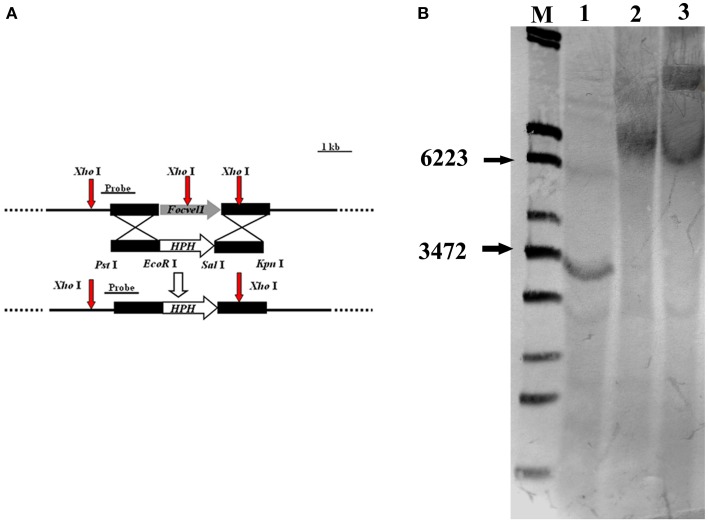
**Schematic representation of the *FocVel1* deletion strategy**. **(A)** *FocVel1* and hygromycin-resistance (*hph*) cassettes are denoted by large gray and white arrows, respectively. Red arrows represent the restriction enzyme sites of *Xho*I. **(B)** Southern blot hybridization analysis. M, λ-EcoT14 I digest; lane 1, genomic DNA preparations of the wildtype strain (Foc-GD); lane 2, the *FocVel1* deletion mutant (Δ*FocVel1*-3); lane 3, the complement strain (Δ*FocVel1*-3C).

To investigate the functions of *FocVel1* gene in *F. oxysporum* f. sp. *cucumerinum*, we generated gene deletion mutants using a homology recombination strategy in the parental *F. oxysporum* f. sp. *cucumerinum* Foc-GD strain (Figure 1A). Among 30 hygromycin-resistant transformants, three mutants (Δ*FocVel1*-3, Δ*FocVel1*-8, and Δ*FocVel1*-15) were randomly selected for further validation. All the putative mutants were identified by PCR and confirmed by Southern blot analysis. When probed with a 1192-bp DNA fragment of *FocVel1*, anticipated bands were present in the wild-type Foc-GD, the deletion mutant Δ*FocVel1*-3 and the complemented strain Δ*FocVel1*-3C, respectively (Figure 1B).

### Expression of *FocVel1* correlates with conidiophores development

**Figure 2:**
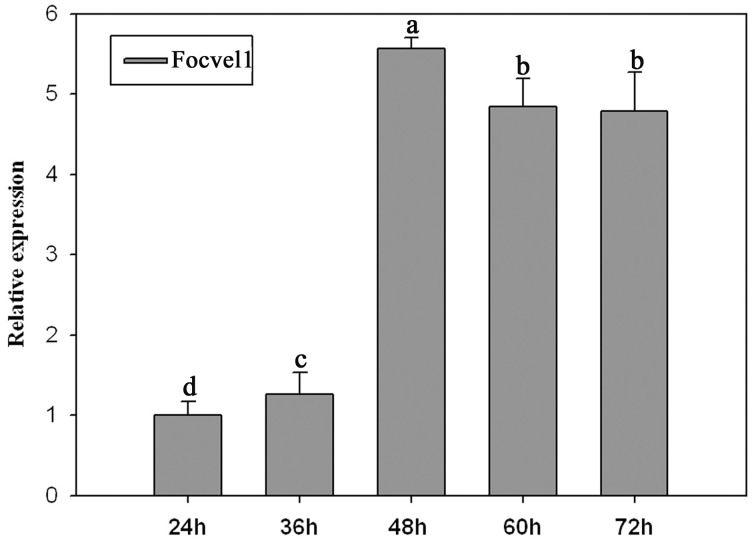
**Relative expression levels of the *FocVel1* in the *F. oxysporum* f. sp. *cucumerinum* wild-type strain Foc-GD at different growth stages**. The *EF-1α* gene was used as the endogenous reference gene for normalization. Line bars in each column denote standard errors from three repeated experiments. Different letters indicate statistically significant differences (*p* < 0.05).

**Figure 3:**
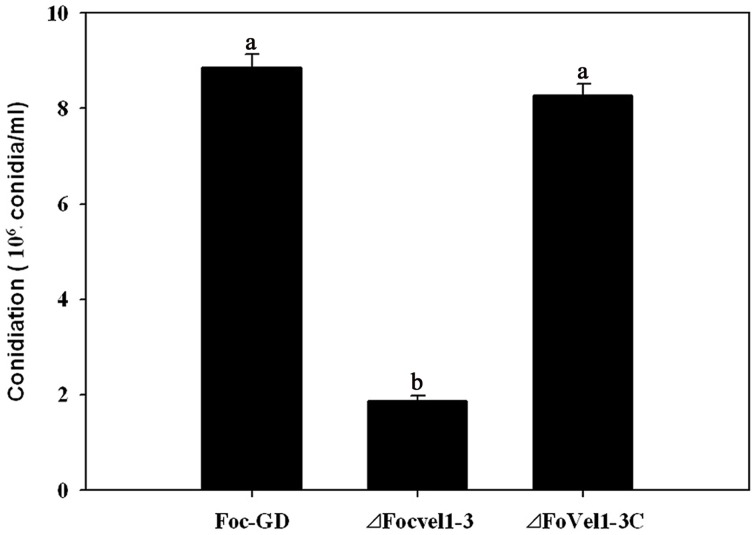
**Conidia were quantified after incubation of the wild-type strain (Foc-GD), *FocVel1* deletion mutant (Δ*FocVel1*-3), and complemented strain (Δ*FocVel1*-3C) in 70 mL of PDB medium for 5 days in a shaker**. Bars represent standard errors from three independent experiments with three technical replicates each. Different letters indicate statistically significant differences (*p* < 0.05).

In several other fungal species, the *VeA* gene has been found to regulate asexual development (Calvo, 2008; Jiang et al., 2011). To study the expression patterns of *FocVel1* in *F. oxysporum* f. sp. *cucumerinum*, we extracted total RNA from strain Foc-GD after culture at different growth stages. Real-time PCR results demonstrated that *FocVel1* expression was low during early growth stages (24 and 36 h, *p* < 0.05), peaked at 48 h, and then remained constant at 60 and 72 h (*p* < 0.05, Figure 2). Conidiophores typically proliferate after 48 h (Zheng et al., 2012). In addition, in PDB medium, the deletion mutant Δ*FocVel1*-3 produced greatly fewer conidia than the wild-type strain or the complemented strain Δ*FocVel1*-3C (*p* < 0.05, Figure 3). Thus, we concluded that *FocVel1* expression may be associated with the development of conidiophores.

### Effects of *FocVel1* on hyphal growth

**Figure 4:**
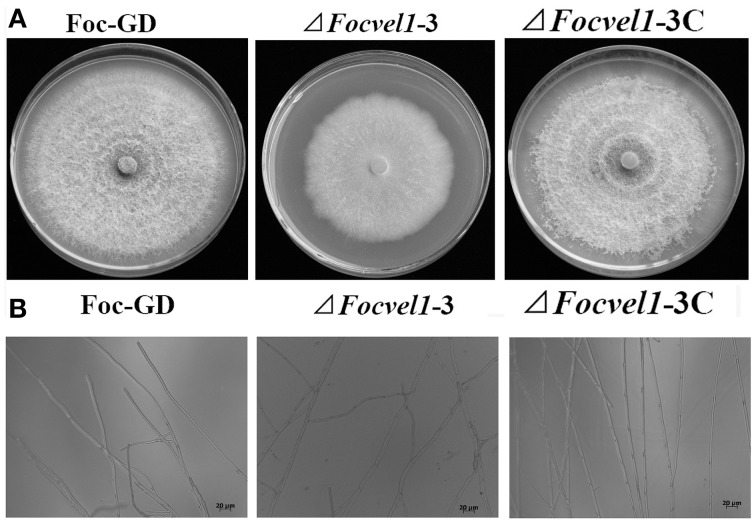
**Effects of *FocVel1* on colony morphology and hyphal morphology**. **(A)** The wild-type strain (Foc-GD), *FocVel1* deletion mutant (Δ*FocVel1*-3), and complemented strain (Δ*FocVel1*-3C) were grown on PDA media for 7 days at 26°C. **(B)** Hyphal morphology and branching patterns of Foc-GD, *ΔFocvel1*-3, and *ΔFocvel1*-3C were examined with optical microscopy. Bar = 20 μm.

The deletion of *FocVel1* dramatically affected the morphology of *F. oxysporum* f. sp. *cucumerinum* colonies on solid media. The Δ*FocVel1*-3 strain displayed a characteristic flat colony phenotype with dramatically reduced aerial mycelium and grew markedly slower than the wild-type progenitor Foc-GD and the complemented strain Δ*FocVel1*-3C on PDA medium (Figure 4A). In addition, colony defects were also observed on MM and CM plates, which eliminated medium-dependent effects (data not shown). Microscopic examination of hyphae revealed that Δ*FocVel1*-3 exhibited a strong increase in hyphal branching relative to the wild-type strain. Moreover, by genetic complementation with the *FocVel1* gene in the complemented strain *ΔFocVel1*-3C, the phenotypic defects were restored when growing on solid media (Figure 4B). These results indicated that the *FocVel1* gene affected the nature of hyphal growth in *F. oxysporum* f. sp. *cucumerinum*.

### Sensitivity of the *FocVel1* deletion mutant to osmotic stress and cell wall-damaging agents

**Figure 5:**
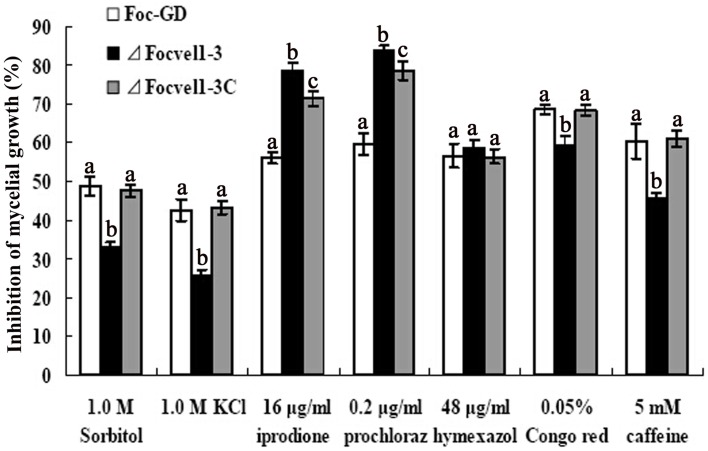
**Sensitivity of the wild-type strain (Foc-GD), *FocVel1* deletion mutant (Δ*FocVel1*-3), and complemented strain (Δ*FocVel1*-3C) to osmotic stresses, fungicides, and cell wall damaging agents**. Osmotic stresses were mediated by the addition of 1.0 M KCl or 1.0 M sorbitol into potato dextrose agar (PDA) medium. The following fungicides were added into PDA: 16 μg/mL iprodione, 0.2 μg/mL prochloraz, or 48 μg/mL hymexazol. Alternatively, the following cell wall-damaging agents were added into PDA: 0.05% Congo red or 5 mM caffeine. Bars denote standard errors from three repeated experiments. Different letters indicate statistically significant differences (*p* < 0.05).

The *VeA* gene deletion mutant has been reported to reduce the hydrophobicity of the cell surface and stabilize osmosis, thereby restoring wild-type hyphal growth and conidiation in mutants partly (Jiang et al., 2011). Therefore, the sensitivity of the *FocVel1* deletion mutant to various stresses was tested too. As shown in Figure 5, compared with the wild-type and complemented strain, Δ*FocVel1*-3 exhibited significantly increased resistance to 1.0 M KCl and 1.0 M sorbitol (*p* < 0.05). The mutant showed increased sensitivity to iprodione (16 μg/mL, *p* < 0.05) and prochloraz (0.2 μg/ mL, *p* < 0.05), but not to hymexazol (48 μg/mL, *p* > 0.05). The restoration of osmotic stabilization shown by Δ*FocVel1*-3 suggested that *FocVel1* may be associated with cell wall organization. To address the hypothesis, we examined the sensitivity of the *FocVel1* mutant to some cell wall-damaging agents, and found that the resistance to such agents (0.05% Congo red or 5 mM caffeine) was greater in Δ*FocVel1*-3 than in the wild-type strain (*p* < 0.05, Figure 5). These results suggested that *FocVel1* was associated with cell wall integrity.

### Effects of *FocVel1* on biofilm development

**Figure 6:**
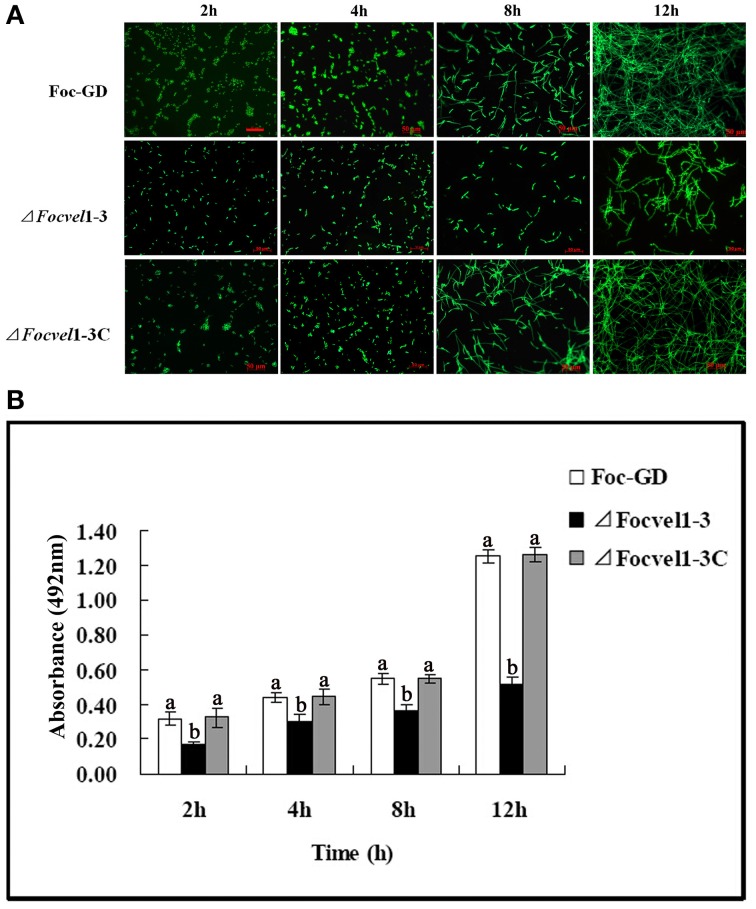
**Comparisons of biofilm formation among the wild-type strain (Foc-GD), *FocVel1* deletion mutant (Δ*FocVel1*-3), and complemented strain (Δ*FocVel1*-3C) on polystyrene strips after incubation for 2, 4, 8, 12 h**. **(A)** Polystyrene strips with biofilms were stained with SYTO-9 and the images were taken using a fluorescence microscope. Scale bar: 50 μm. **(B)** The biofilms were quantified by XTT reduction assay. Bars represent standard errors from three independent experiments with three technical replicates each. Different letters indicate statistically significant differences (*p* < 0.05).

In an earlier study (Li et al., 2014), we showed that *F. oxysporum* f. sp. *cucumerinum* can form biofilms in flat-bottomed polystyrene microtitre plates. Furthermore, in several fungal species, the velvet protein has been shown to be involved in the regulation of diverse cellular processes (Jiang et al., 2011). Here, we tested whether the *FocVel1* participated in the process of *F. oxysporum* f. sp. *cucumerinum* biofilm formation. After incubation of the wild-type and complemented strains with a standardized spore suspension, we found that spore aggregates adhered to the polystyrene surface (0–2 h) and began to swell and germinate within 2–4 h. Hyphae were observed within 4–8 h, and mycelia monolayers or hyphal networks appeared by 8–12 h. In contrast, in the *ΔFocVel1* strain group, fewer disperse spores and a hyphal monolayer were observed to be attached to the polystyrene surface after 2 or 12 h of incubation. With continuous incubation, hyphal networks of the Δ*FocVel1* strain were still thinner than those of the wild-type and complemented strains (data not shown). In other words, deletion of *FocVel1* not only reduced the number of aggregative spores but also led to a delay in conidial germination (Figure 6A). In parallel, we quantified the biofilms of wild-type, mutant, and complemented strains after incubation at 28°C by XTT reduction assays at different time points. As shown in Figure 6B, quantification of the biofilms of wild-type and complemented strains were approximately 1.23−, 0.52−, 0.69−, and 1.56-fold that of Δ*FocVel1*-3 after being incubated for 2, 4, 8, or 12 h, respectively (*p* < 0.05).

**Figure 7:**
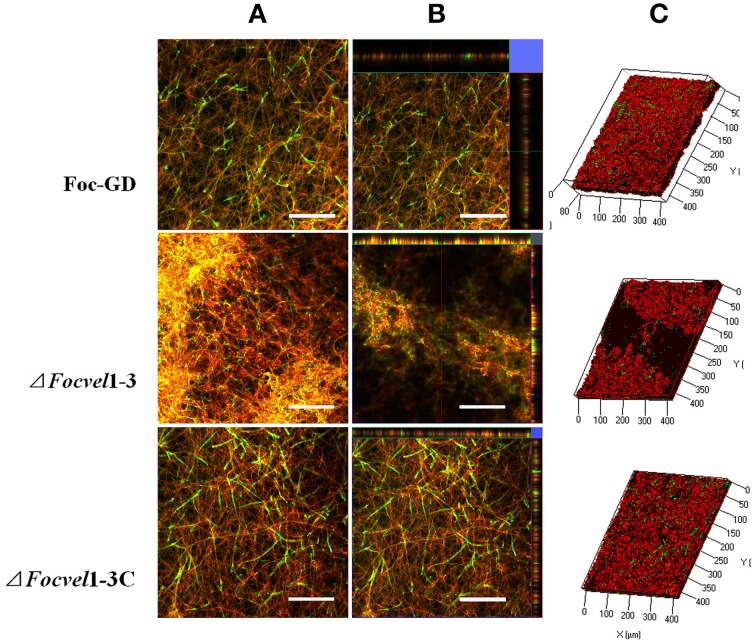
**CLSM images of biofilm formation by the wild-type strain (Foc-GD), *FocVel1* deletion mutant (Δ*FocVel1*-3), and complemented strain (Δ*FocVel1*-3C) at 48 h**. Cell wall-like polysaccharides and heat-killed biofilm cells were marked with green and red fluorescence by ConA and PI, respectively. **(A)** Double-stained mature biofilms. **(B)** Lateral views of the three-dimensional images. **(C)** Three-dimensional reconstruction of biofilms after dual staining. Scale bar: 100 μm.

The architecture of mature biofilms was observed using a Zeiss LSM710 CLSM as described previously (Chandra et al., 2008; Mukherjee et al., 2012). As expected, there was an obvious morphological alteration in the biofilm of the *FocVel1* mutant. The mutant Δ*FocVel1*-3 showed significant defects in the thin biofilms that formed on the polystyrene surface, exhibiting heterogeneous hyphae and EPS production (Figure 7). Although some aggregative hyphae formed protrusions on the surface of Δ*FocVel1*-3 biofilms, most of the area of the structure was very loose. In contrast, the wild-type and complemented strains showed a highly organized architecture, with red hyphal cells interwoven into the structure and green EPS materials visible (Figure 7). These data suggested that *FocVel1* may affect initial spore adhesion and subsequently cause deficient fungal biofilm formation in *F. oxysporum* f. sp. *cucumerinum*.

### *FocVel1* is essential for virulence of *F. oxysporum* f. sp. *cucumerinum*

**Figure 8:**
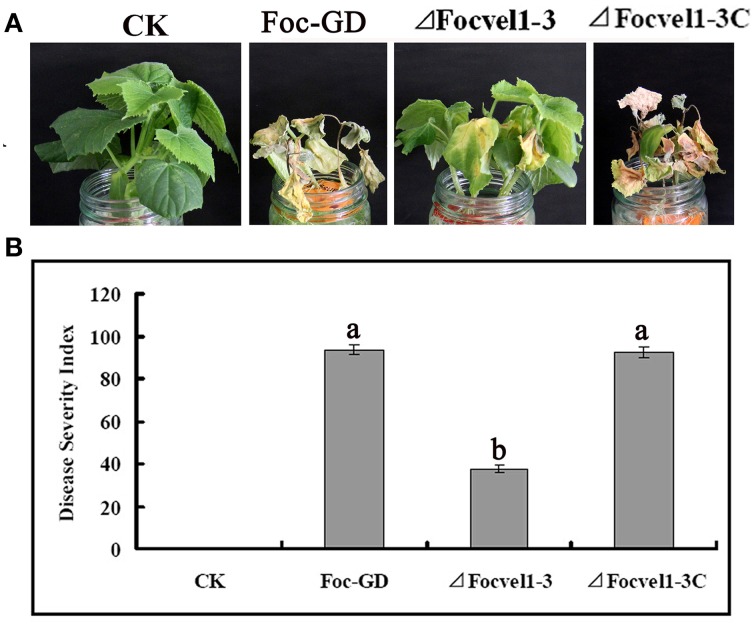
**Effects of *FocVel1* on the virulence of *Fusarium oxysporum* f. sp. *cucumerinum***. Cucumber seedlings were inoculated with 1 × 10^6^ conidia/mL from each strain. **(A)** DSIs in cucumber seedlings 15 days after inoculation. **(B)** Symptoms of cucumber plants were photographed 15 days after inoculation. CK, Control plants in good health. Bars represent standard errors from three independent experiments with three technical replicates each. Different letters indicate statistically significant differences (*p* < 0.05).

To compare the virulence of the deletion mutant Δ*FocVel1*-3, the wild-type strain Foc-GD and the complemented strain (Δ*FocVel1*-3C), the susceptible cucumber cultivar (*Cucumis sativus* L. 9930) was inoculated with a standardized spore suspension of 1 × 10^6^ conidia/mL. Cucumber seedlings inoculated with conidia of the wild-type or complemented strain showed progressive wilt symptoms and usually died at 15dpi and the DSIs of Fusarium wilt were 93.7 and 92.3, respectively (Figure 8A). In contrast, under the same conditions, plants inoculated with the Δ*FocVel1*-3 mutant displayed a significantly lower DSI (37.3, *p* < 0.05), and most of the plants inoculated with this mutant survived the assay or developed only mild disease symptoms (Figure 8B). These results demonstrated that the disruption of *FocVel1* reduced the virulence of *F. oxysporum* f. sp. *cucumerinum* on cucumbers.

## Discussion

In this study, we examined the roles of the *FocVel1* gene in the plant pathogenic fungus *F*. *oxysporum* f. sp. *cucumerinum*, which causes destructive CFW. *FocVel1* was highly expressed during conidiophore development, and disruption of the *FocVel1* gene led to several phenotypic defects. Moreover, the mutant *ΔFocVel1* exhibited impaired virulence in cucumber seedlings. Thus, our data indicated that *FocVel1* played a critical role in the regulation of various cellular processes and pathogenicity in *F. oxysporum* f. sp. *cucumerinum*.

The velvet complex containing VeA and several other regulatory proteins, plays an important role in fungal growth, colony morphology, development, and secondary metabolism in several filamentous fungi (Li et al., 2006; Jiang et al., 2011). Certain variations in this role have been observed in many fungi, such as in the genera *Aspergillus* and *Fusarium* (Li et al., 2006; Calvo, 2008; Wiemann et al., 2010; Jiang et al., 2011). Bioinformatics analyses (Broad Institute Fungal Genome Initiative, http://www.broadinstitute.org/) have revealed that fungal VeA proteins contain conserved N-terminal and variable C-terminal regions, which may be responsible for the conserved and species-specific functions of VeA, respectively. Here, we found that some of the phenotypes in the *FocVel1* mutant were similar to those reported in other fungi, while others were novel to *F. oxysporum* f. sp. *cucumerinum*.

In *F. graminearum*, *FgVEA* deletion causes delays in conidial germination, however, conidia from the *FgVEA* deletion mutant germinate to form normal, unbranched germ tubes (Jiang et al., 2011). In *Aspergillus nidulans* and *Neurospora crassa*, deletion of *veA* leads to a significant increase in conidial formation (Kim et al., 2002; Bayram et al., 2008). In contrast, a significant reduction in conidiation occurs following deletion of *veA* in *Penicillium chrysogenum* (Hoff et al., 2010) and *Aspergillus fumigatus* (Krappmann et al., 2005), and similar results have been observed in *vel1* mutants of *F. verticillioides* (Li et al., 2006) and *F. fujikuroi* (Wiemann et al., 2010). In current study, we also observed that the *FocVel1* deletion mutant of *F. oxysporum* f. sp. *cucumerinum* produced significantly fewer conidia than that of the wild-type strain. Moreover, the *FocVel1* mutants showed a strong increase in hyphal branching. These results strongly indicated that the roles of VeA in asexual differentiation varied significantly among different fungal species.

The fungal cell wall is required for maintaining cell integrity, and it plays an important role in many important processes. In addition, the cell wall provides the cell with sufficient mechanical strength to withstand changes in osmotic pressure and other environmental stresses (Gelis et al., 2012; Zheng et al., 2013). Previous studies have shown that the *A. nidulans velB* mutant increases the sensitivity of the strain to H_2_O_2_ and UV light due to impaired accumulation of trehalose (Bayram et al., 2008). *FgVEA* deletion in *F. graminearum* has been shown to increase tolerance to osmotic stress mediated by NaCl and KCl, consistent with the observation glycerol accumulation is higher in the mutant than in the wild-type strain (Jiang et al., 2011). In the current study, we found that the *FocVel1* deletion mutant exhibited increased resistance to various stress agents, including sorbitol, KCl, Congo red, and caffeine. These results suggested that the increased resistance to cell wall-damaging agents may result from the increased accumulation of cell wall materials. Thus, *FocVel1* might have an important role in maintaining normal cell wall composition and integrity.

*F. oxysporum* was ranked fifth in a recent survey of the top 10 fungal plant pathogens and could produce vascular wilt disease in over 100 different plant species (Dean et al., 2012). *F. oxysporum* grows inter- and intracellularly through the cortex before entering the vascular bundles, and then uses the xylem vessels as avenues to grow upwards into the stem and colonize the plant, provoking the characteristic wilt symptoms (Perez-Nadales and Di Pietro, 2011). Our previous studies have shown that the pathogenic *F. oxysporum* f. sp. *cucumerinum* strain Foc-GD can form biofilms *in vitro* (Li et al., 2014). Fungal biofilms might be involved in the pathogenesis of localized as well as invasive diseases caused by fungi of medical relevance to humans, such as *A. fumigatus* (Harding et al., 2009) and *Candida albicans* (Imamura et al., 2008).

In this study, in the process of *F. oxysporum* f. sp. *cucumerinum* biofilm formation, we observed that conidia of the Δ*FocVel1* mutant were less adherent on the polystyrene surface and exhibited delayed conidial germination in static culture. Moreover, the Δ*FocVel1* mutant exhibited impaired biofilms that were markedly attenuated and loose after 24 h incubation, and heterogeneous hyphae were attached to the polystrene surface. In yeast, biofilm formation has been shown to begin with adherence of the cells to a substrate. Biofilm adhesins and several other cell wall proteins play key roles in adherence (Yu et al., 2012). Our study showed that conidia of the Δ*FocVel1* mutant strain reduced adherence to surfaces and were defective in biofilm formation. Similarly, the result was consistent with quantitative analysis by XTT reduction assay in polystyrene microtiter plates. Thus, the defects in in biofilm formation in the mutant were likely to be associated with a disturbance in *FocVel1* function.

Phenotypic characterization of the *FocVel1* deletion mutant showed that *FocVel1* was required for the virulence of *F. oxysporum* f. sp. *cucumerinum*. The impairment in virulence in the *FocVel1* deletion mutant appeared to be due to defects in multiple regulatory functions. First, the deletion of *FocVel1* caused a delay in conidial germination and suppression of mycelial growth. Second, VeA proteins have been shown to be involved in the production of secondary metabolites, including the mycotoxins deoxynivalenol, beauvericin, and fusaric acid in several fungi during infection (López-Berges et al., 2013). In addition, the hydrophobic properties of the cell surface and the normal fungal cell wall have been shown to the important for viability and virulence (Muller et al., 2002). In our current study, our data demonstrated that a series of defects resulting from the deletion of *FocVel1*, including hyphal growth, cell wall integrity, and biofilm formation, may have contributed to the impaired in virulence observed in the mutant strain on cucumber seedlings.

In conclusion, our study demonstrated that *FocVel1* is essential for physiological processes, such as asexual development, hyphal growth, and cell wall integrity in *F. oxysporum* f. sp. *cucumerinum*, and it contributed to the attenuated virulence observed in cucumber seedlings. Moreover, *FocVel1* was required for adherence to inorganic substrates as well as for biofilm formation. To our knowledge, this is the first report showing that *VeA* is involved in biofilm formation in a fungal plant pathogen. However, whether the *F. oxysporum* f. sp. *cucumerinum* strain could form biofilms in the vascular bundles of the hosts is not yet known, and the role of biofilm formation in the infection process is not well defined, Therefore, it will be interesting to elucidate relationships between biofilm formation and pathogenic mechanisms in *F. oxysporum*, which may improve our understanding of the biology of *F. oxysporum* f. sp. *cucumerinum*.

### Conflict of interest statement

The authors declare that the research was conducted in the absence of any commercial or financial relationships that could be construed as a potential conflict of interest.

## Supplementary material

Figure 1 **Multiple sequence alignment of *FocVel1* with other characterized VeA homologous sequences**. Dark highlights indicate that the residues were conserved in all of the VeAs compared, whereas other colors denote sequences only conserved in some VeAs. The aligned sequences were from: *Ncve-1* (accession number: XP_957154) from *Neurospora crassa*, *PcVeA* (accession number: CAP92389) from *Penicillium chrysogenum*, *AcVeA* (accession number: CAL68582) from *Acremonium chrysogenum*, *AfVeA* (accession number: XM_747526) from *Aspergillus fumigatus*, *ApVeA* (accession number: AY445513) from *Aspergillus parasiticus*, *AnVeA* (accession number: U95045) from *Aspergillus nidulans*, *FgVeA* (Accession number: JN635273) from *Fusarium graminearum*, *Ffvel1* (accession number: FN548142) from *Fusarium fujikuroi*, and *Fv_VE1* (accession number: DQ274059) from *Fusarium verticillioides*.
