# Supplementary figures and images for: FocVel1 influences asexual production, filamentous growth, biofilm formation, and virulence in Fusarium oxysporum f. sp. cucumerinum
Source: Front Plant Sci. 2015 May 6;6:312. doi: 10.3389/fpls.2015.00312 (PMC4422011; doi:10.3389/fpls.2015.00312)

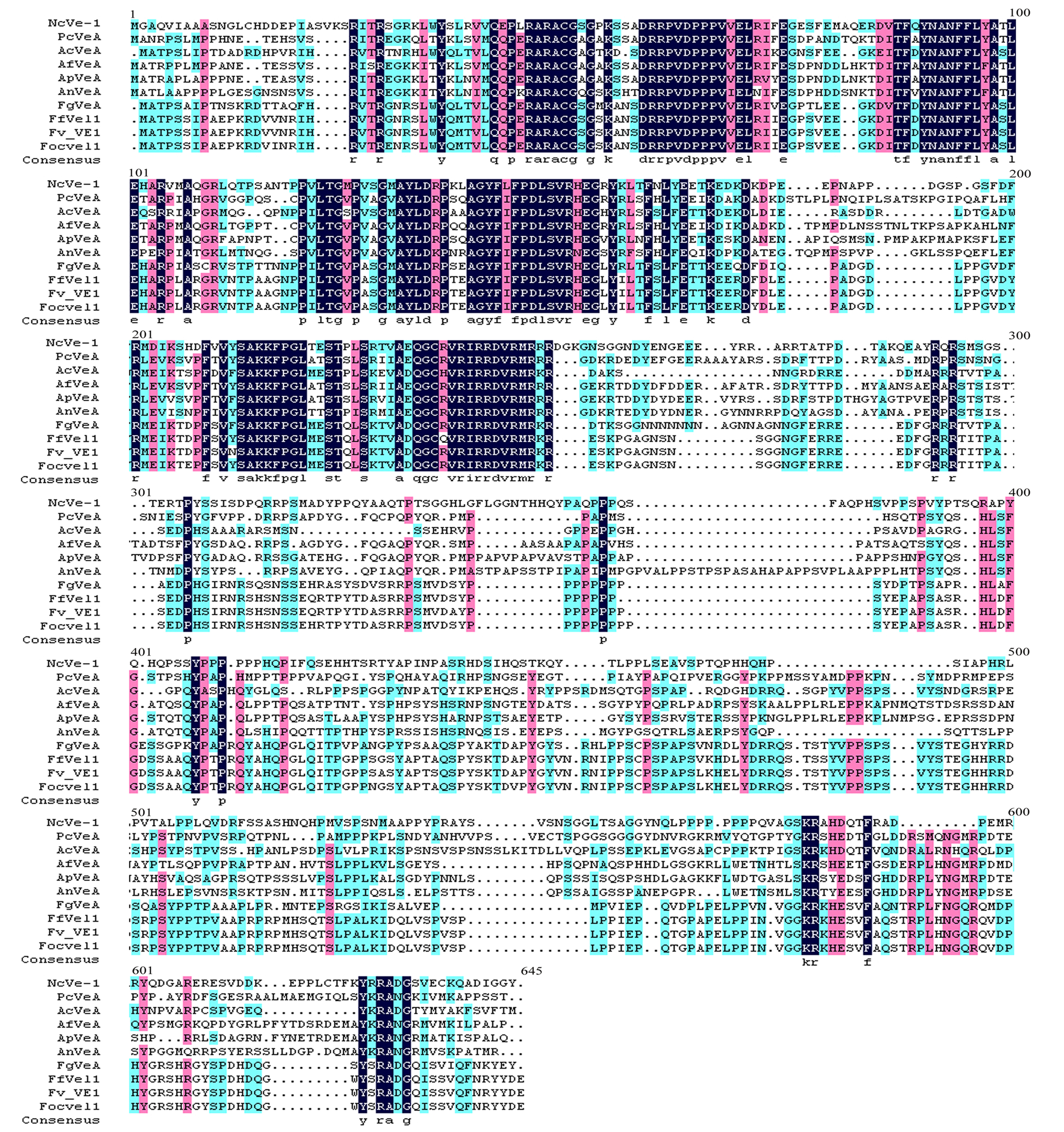

Supplement: Figure 1 — Multiple sequence alignment of FocVel1 with other characterized VeA homologous sequences. Dark highlights indicate that the residues were conserved in all of the VeAs compared, whereas other colors denote sequences only conserved in some VeAs. The aligned sequences were from: Ncve-1 (accession number: XP_957154) from Neurospora crassa, PcVeA (accession number: CAP92389) from Penicillium chrysogenum, AcVeA (accession number: CAL68582) from Acremonium chrysogenum, AfVeA (accession number: XM_747526) from Aspergillus fumigatus, ApVeA (accession number: AY445513) from Aspergillus parasiticus, AnVeA (accession number: U95045) from Aspergillus nidulans, FgVeA (Accession number: JN635273) from Fusarium graminearum, Ffvel1 (accession number: FN548142) from Fusarium fujikuroi, and Fv_VE1 (accession number: DQ274059) from Fusarium verticillioides. [file Image1.JPEG]
